# Effect of Bioptron light therapy on dryness of eyes in postmenopausal women: a randomized controlled trial

**DOI:** 10.1007/s10103-026-04807-6

**Published:** 2026-02-04

**Authors:** Ehab Mohamed Elsayed Saad, Sara Magdy Ahmed, Amel Mohamed Yousef, Elham Shahat Hassan

**Affiliations:** 1 Department of Ophthalmology, Faculty of Medicine, Benha University, Benha, Egypt; 2 Department of Physical Therapy for Women’s Health, Faculty of Physical Therapy, Cairo University, Cairo, Egypt; 3 Department of Physical Therapy for Women’s Health, MSA University, Giza, Egypt

**Keywords:** Bioptron, Photo biomodulation, Dry eye, Postmenopausal women

## Abstract

To examine the short-term therapeutic effects of Bioptron light therapy (BLT) on dry eye disease (DED) in postmenopausal women. Sixty postmenopausal women diagnosed with DED, aged 50–62 years with a body mass index ≤ 30 kg/m², were randomly assigned to two equal groups. The Bioptron group (*n* = 30) received BLT for 10 min, twice weekly, along with an educational program for four weeks. The control group (*n* = 30) received the same educational program only. Outcomes were measured using Schirmer’s test (Schirmer I) for basal tear secretion, Tear Break-Up Time (TBUT) for tear film stability, and the Dry Eye-related Quality of Life Score (DEQS) for quality of life (QOL). Both groups exhibited significant improvements post-treatment compared to pre-treatment in all variables, with greater percentage changes from baseline in the BLT group. Between-group comparisons revealed significantly higher improvements in the BLT group: Schirmer’s test (Schirmer I) improved by MD = 8.7 (95% CI: 7.64–9.76; *p* = 0.001), TBUT by MD = 2.97 (95% CI: 1.7–4.3; *p* = 0.007), and DEQS decreased by MD = − 24.1 (95% CI: −37 to − 17.1; *p* = 0.002). Compared with standardized education alone, adjunctive Bioptron light therapy significantly enhanced tear secretion, tear film stability, and patient-reported symptoms, suggesting its potential as an effective non-pharmacological option for managing dry eye in postmenopausal women. The trial was registered at Clinical Trials.gov (Identifier: NCT05964673)

## Introduction

Dry eye disease (DED) represents an escalating global health concern, that impairs visual function and quality of life (QOL), and imposes notable socio-economic costs [1]. DED involves a breakdown in ocular surface homeostasis, leading to instability of the tear film, high tear osmolarity, inflammatory responses, and ocular surface damage. Such physiological disturbances manifest as symptoms such as visual discomfort while reading or watching television, difficulty driving at night, blurred vision, photophobia, and sensations of burning, itching, and redness [2]. DED is divided according to pathophysiological mechanisms into aqueous-deficient dry eye (ADDE) and evaporative dry eye (EDE), categories that guide diagnosis and treatment decisions. Factors like lid laxity, reduced blink reflex, systemic inflammation, medication use, and autoimmune disorders contribute to age-related tear decline [3].

DED disproportionately affects women, especially postmenopausal, due to hormonal shifts and aging-related ocular changes [4]. Menopause is closely linked to DED due to hormonal disruptions which destabilize the ocular surface [5]. Sex hormones — including estrogens, androgens, and progestogens — play a regulatory role in the function of the lacrimal and meibomian glands. These glands are essential components of the lacrimal functional unit (LFU), which maintains tear film stability and ocular surface health [6]. Estrogen receptor mRNA has been identified in the lacrimal gland, meibomian glands, eyelids, conjunctiva, corneal tissue, and other anterior ocular structure [7]. Hormonal imbalance during menopause alters the secretion of watery components and glycoprotein mucins from tear-producing glands and conjunctival mucin-secreting cells, and lipids from the meibomian glands, compromising tear film integrity [8].

DED diagnosis requires full ophthalmologic evaluation comprising visual acuity, refraction, and assessment of the eyelids and meibomian glands [9]. Patient-reported outcome (PRO) tools like the Ocular Surface Disease Index (OSDI), Standardized Patient Evaluation of Eye Dryness (SPEED), and Dry Eye Questionnaire (DEQ-5) are commonly used [10]. Diagnostic tests include slit-lamp biomicroscopy, tear breakup time (TBUT), Schirmer’s test, tear film imaging, osmolarity testing, ocular surface cultures, and evaluation of matrix metalloproteinase-9 (MMP-9) [11].

DED treatment follows a tiered approach. First-line therapy involves education, environmental adjustments, identification of triggers, artificial tears, lid hygiene, and oral essential fatty acids [12]. Second-line includes tea tree oil for Demodex, non-preserved lubricating agents, punctal plugs, moisture-retaining goggles, nocturnal ointments, meibomian gland expression, intense pulsed light (IPL), and topical drugs. Third-line options are oral secretagogues, autologous or allogenic serum drops, and specialized contact lenses. Surgery is considered a last resort. Despite their use, lubricants offer only temporary relief and may take time to show effect, underscoring the need for new therapies [13]. Recent clinical studies of low-level light therapy (LLLT), including LED and IPL modalities, have reported improvements in tear film stability and dry eye symptoms, implying photobiomodulation may support ocular surface homeostasis [14]. Furthermore, systematic reviews of IPL therapy highlight significant efficacy in meibomian gland dysfunction–related dry eye, reinforcing the therapeutic promise of light-based interventions [15].

Polarized light therapy (PLT), a form of photobiomodulation therapy (PBMT), has emerged as a novel intervention. Bioptron Light Therapy (BLT) utilizes a broad-spectrum polarized polychromatic, non-coherent light (350–3400 nm) that penetrates tissues up to 5 cm. This mechanism, based on the principle of photobiomodulation, influences cellular metabolism, microcirculation, and reduces oxidative stress [16, 17]. Specifically, PBMT has been shown to promote cellular repair, reduce inflammation through immunomodulatory effects, and support essential cellular functions like molecular transport and energy conversion [16, 18]. On the ocular surface, these mechanisms may stabilize the tear film, improve meibomian gland function, and reduce inflammatory mediators [18]. While PLT has been widely applied in dermatology, wound healing, and pain management, its potential ophthalmic applications have not been fully investigated. To our knowledge, no previous randomized controlled trial has evaluated PLT for DED, highlighting the novelty of this investigation as non-pharmacological therapy for postmenopausal women with DED. The aim of this study was to evaluate the short-term effect of BLT, when added to a standardized educational program, on DED in postmenopausal women. The primary objective was to assess improvement in basal tear production using the Schirmer I test. Secondary objectives included evaluating tear film stability (TBUT) and dry-eye–related quality of life (DEQS) over a 4-week period.

## Materials and methods

This study was a single-center, prospective, randomized, single-blind, parallel-group exploratory clinical trial (RCT) conducted to evaluate the short-term efficacy and safety of Bioptron light therapy (BLT) as an adjunct to a standardized educational program in postmenopausal women with dry eye disease (DED). The person-centered study designs have gained attention in recent years. Badian et al. [19] proposed the person-centered randomized controlled trial (PC-RCT), offering an innovative perspective for study design. Ethical approval was obtained from the Faculty of Physical Therapy, Cairo University Ethics Committee (P.T.REC/012/004549), and the trial was registered at ClinicalTrials.gov (Identifier: NCT05964673). The intervention period spanned from May to July 2023. As this was a preliminary, hypothesis-generating study, the design prioritized feasibility and detection of a clinically meaningful signal to inform larger, definitive trials.

### Participants and eligibility

Sixty-six postmenopausal women were recruited from outpatient ophthalmology clinic, Benha University teaching hospital, Qalyubia. The recruitment process consisted of conducting direct interviews with women.

## Eligibility criteria

Participants were evaluated based on the following eligibility criteria: all participants were postmenopausal (stoppage of menses for at least 1 year following last menstruation) naturally; their age ranged from 50 to 62 years, and they complained of dry eye symptoms including discomfort, dryness, itching, burning, sensitivity to light, tearing, discomfort, grittiness, and visual disturbances ranging from mild to moderate and severe. Participants were enrolled based on the TFOS DEWS II diagnostic criteria for dry eye disease and on symptomatic complaint of DED. Baseline evaluation included Schirmer I (with topical anesthesia) and TBUT. Subtyping of DED was not performed in this study. Although factors such as meibography and lipid layer grading can inform subtype classification, our primary focus was on evaluating the overall efficacy of BLT as an adjunct intervention. The trial did not include formal etiologic subtyping into aqueous-deficient (ADDE), evaporative (EDE), or mixed types using advanced diagnostic tools such as meibography, lipid layer assessment, or tear osmolarity. Consequently, the study population may have included patients with mixed underlying etiologies. Although Schirmer’s test (Schirmer I) and TBUT were assessed at baseline, these measures alone are insufficient for precise subtyping. All enrolled participants, however, fulfilled the TFOS DEWS II diagnostic criteria for dry eye disease. We specifically targeted postmenopausal women because hormonal decline, particularly estrogen and androgen deficiency, is strongly associated with lacrimal gland dysfunction, meibomian gland atrophy, and higher prevalence of DED. This group therefore represents an ideal population to explore novel interventions such as PLT.

## Exclusion criteria

After a detailed medical history was documented, participants with allergy response to fluorescein, smoking participants, and systemic disease were excluded since it can cause dry eye syndrome and reduce the skin’s and mucous membrane’s ability to regenerate. Eyes were thoroughly examined for signs of disease including chronic blepharitis, meibominitis, or any other eye infection, as well as for signs of injury or past surgical treatment. Participants were permitted to continue using preservative-free carboxymethylcellulose drops (*Polyfresh Extra SDU*, Orchidia Pharmaceutical, Egypt), which were standardized across groups. The study did not include participants who were taking hormone replacement therapy.

## Randomization and blinding

**Fig. 1:**
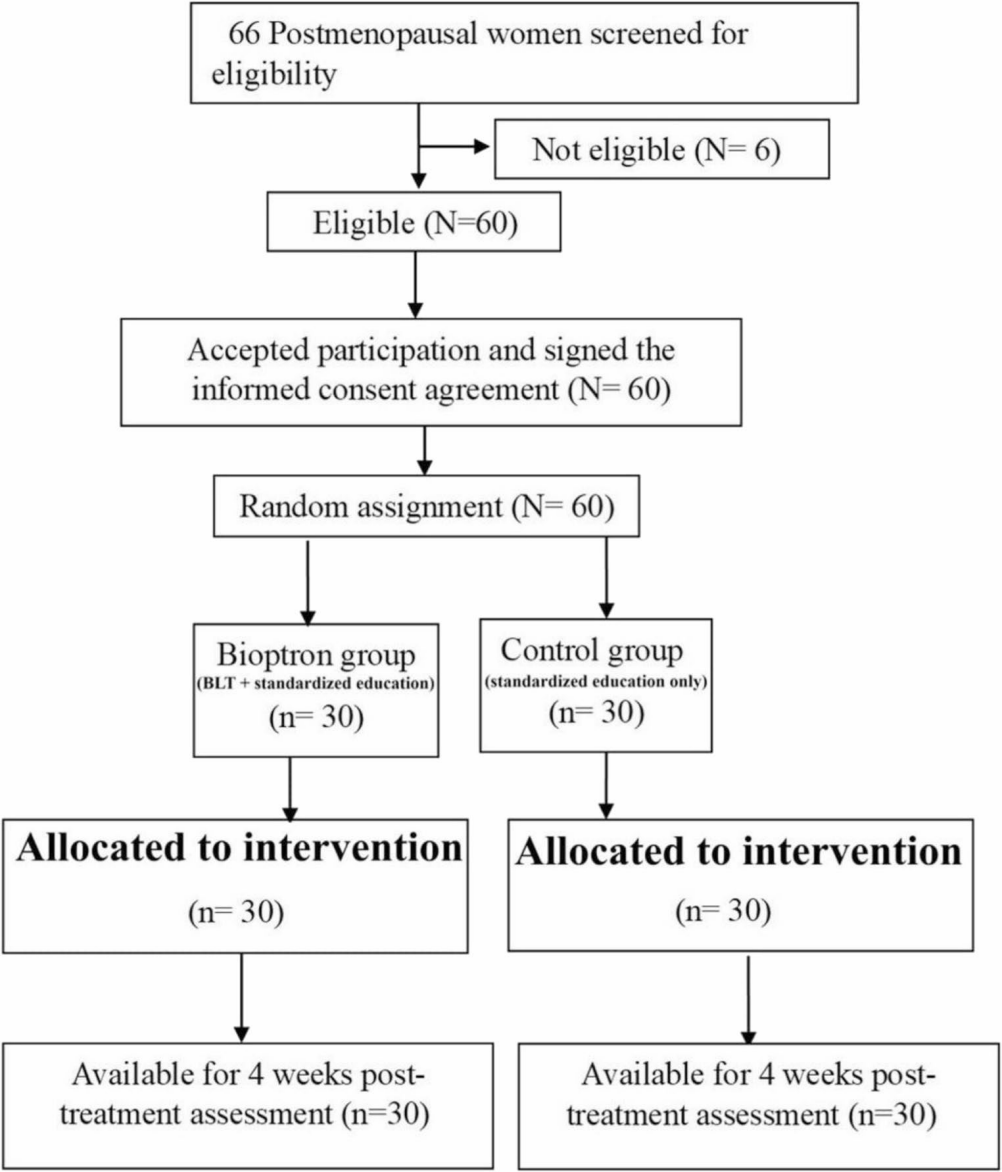
Consort flow diagram illustrating participant enrollment, randomization, allocation, follow-up, and analysis. BLT = Bioptron light therapy

Participants were randomly allocated in a 1:1 ratio to either the Bioptron group (A) (*n* = 30) or the control group (B) (*n* = 30). Randomization was performed after baseline evaluation using a simple randomization sequence generated by Research Randomizer, an online tool for generating random assignments (https://www.randomizer.org/). No block randomization or stratification was applied; however, initial demographic data and clinical profiles were comparable between groups (Table 1). Group assignments were prepared on sequentially numbered index cards by an independent researcher not involved in the trial to ensure allocation concealment. The cards were sealed in opaque envelopes, which were opened by a physiotherapist during intervention sessions to determine group assignment. Baseline and post-treatment evaluations (week 4) were conducted by an ophthalmologist who was masked to group allocation (Fig. 1). This was a single-blind study in which outcome assessors were blinded to group allocation. Participants were aware of their treatment assignment, but the investigators performing Schirmer, TBUT, and CFS grading, as well as those analyzing questionnaires, were masked to intervention status to reduce bias.

**Table 1:** Comparison between Bioptron and control group according to demographic data (*N* = 60)

Demographic data	Bioptron Group (*n* = 30)	Control Group (*n* = 30)	t-value	*p*-value
Age (years)	56.7 ± 3.3	56.6 ± 3.3	0.1	0.94
Weight (kg)	75 ± 6.4	75.1 ± 5	−0.1	0.91
Height (cm)	164.3 ± 4.5	165.3 ± 3.5	−0.03	0.98
BMI (kg/cm ^2^ )	27.8 ± 2	27.82 ± 1.9	−0.1	0.89
Years of post-menopausal	7.9 ± 1.6	8.3 ± 1.2	−1.2	0.42

Data were expressed as mean ± Standard deviation. N number. P probability, BMI body mass index, Kg kilogram, Cm centimeter, kg/cm^2^kilogram per centimeter square

## Consent process

A comprehensive explanation of the study’s purpose, procedures, and rationale was provided to ensure participant trust and cooperation. Participants were assured of confidentiality and informed of their right to withdraw at any point. All participants received a standardized, comprehensive verbal and written explanation of the study procedures, potential risks and benefits, and the randomization process prior to providing written informed consent. Following consent and baseline assessment, participants were randomized. No participant refused randomization or requested reassignment after allocation. Any questions or hesitations expressed by participants were addressed by the study team at recruitment; statements of participants’ second thoughts or withdrawal reasons are summarized in Fig. 1 (flowchart). We also note that person-centered approaches to communication about randomization may help reduce decisional uncertainty [20].

### Interventions

The intervention was administered twice weekly for four weeks, resulting in a total of eight sessions. A skilled ophthalmologist with 7 years of clinical experience conducted an educational program for both groups. Alongside the education program, the Bioptron group also received BLT for 10 min 2 sessions a week for 4 weeks. Participants in both groups received identical standardized education including preservative-free carboxymethylcellulose eye drops (*Polyfresh Extra SDU*, Orchidia Pharmaceutical, Egypt) and omega-3 supplementation. Adherence was verbally reinforced at each visit, but compliance was not formally recorded. Participants were monitored for adverse events (ocular discomfort, redness, photophobia, skin irritation) at each session and during follow-up. Safety was assessed systematically at each visit by recording any local or systemic reactions.

### Bioptron light therapy

The Bioptron group participants were treated using the Bioptron Pro1 device manufactured by AG, Switzerland (100–240 V-,50/60HZ,75 W). This light therapy device was used for the irradiation of both eyes simultaneously in all participants in group (A) with the following output characteristics: wavelength = 350–3400 nm; polarization degree exceeding 95% (590–1550 nm); power density = 40 mW/cm^2^; light energy per minute = approximately 2.4 J/cm^2^. The sessions were held during the day in a room with a temperature of 26 degrees (air conditioned), and low illumination. Participants were positioned in supportive chairs, maintaining closed eyes with pre-sanitized eyelid regions, intermittently blinking. The Bioptron device was positioned perpendicular to the treatment area at a 10 cm working distance, with 10-minute exposure duration [21].

### Patient education program

All participants in both groups (A and B) received a standardized 4-week education program. The content included guidance on reducing screen time, practicing blinking awareness exercises, maintaining a cool and humid indoor environment, and avoiding air drafts. Participants were instructed to use preservative-free artificial tears (Blink® sodium hyaluronate) twice daily, minimize contact lens wear, ensure adequate hydration, limit caffeine intake, and incorporate omega-3 polyunsaturated fatty acids through diet or supplementation [22]. Although lifestyle modifications may influence the severity of dry eye disease, these recommendations were applied equally to both groups to minimize potential confounding effects. All the outcome measures were conducted and assessed by the ophthalmologist.

#### Study outcomes

The primary outcome of this exploratory study was basal tear secretion, measured using the Schirmer I test. Secondary outcomes included tear film stability, assessed by tear break-up time (TBUT), and dry-eye-related quality of life, evaluated using the Dry Eye-related Quality of Life Score (DEQS). The aim was to evaluate the short-term effect of BLT, added to a standardized educational program, on DED in postmenopausal women over a 4-week period. Given the exploratory nature of the study, formal sample size recalculation was not performed, and all findings are presented as preliminary short-term data to inform future larger trials.

#### Basal tear secretion

Basal tear secretion was assessed using Schirmer’s test (Schirmer I) in both eyes simultaneously for all participants, pre- and post-treatment. Required materials included Whatman filter paper No. 41, topical anesthetic eye drops, and a stopwatch. Each subject was seated comfortably in the examination room. After hand sanitization, the ophthalmologist instilled one drop of Benoxinate hydrochloride (4.4 mg/mL) into the inferior fornix of each eye. Ten minutes later, Whatman strips (35 mm × 5 mm), labeled “L” (left) and “R” (right), were folded at a 90° angle and positioned between the palpebral and bulbar conjunctiva, avoiding contact with the lashes or cornea, as the patient looked upward while the lower lid was gently retracted. Following placement, participants were instructed to gently close their eyes without squeezing for 5 min. At the end of this period, the subject reopened their eyes and looked upward for strip removal. Schirmer’s test (Schirmer I) results were determined by measuring the length of the moistened area on each strip, reflecting tear secretion over five minutes. Values were interpreted as follows: 0–5 mm indicated severe DED, 5–10 mm moderate, 10–15 mm possible, and > 15 mm normal tear production [23, 24].

#### Tear film stability

Tear film stability was assessed utilizing the Tear Break-Up Time (TBUT) test, performed sequentially on both eyes of all participants before and after the intervention by an experienced ophthalmologist. The required tools a fluorescein strip, slit-lamp biomicroscope with cobalt blue filter, and a stopwatch were prepared in advance. Each participant was seated comfortably in a darkened examination room. A fluorescein strip was positioned in the inferior fornix, and the participant was directed to blink multiple times to ensure even dye distribution on the tear film. Afterward, they were told to avoid blinking, and the duration between the last full blink and the first visible disruption of the tear film was timed under cobalt blue illumination. The procedure was then repeated for the contralateral eye.

For interpretation, TBUT values were classified as follows: ≥10 s = normal tear film stability, 5–9 s = moderate instability, and < 5 s = severe instability. Schirmer I values were categorized as ≤ 5 mm = severe deficiency, 6–10 mm = moderate deficiency, 11–15 mm = mild/possible deficiency, and > 15 mm = normal. For reference, Schirmer I values < 10 mm indicated abnormal basal tear secretion, and TBUT < 10 s indicated tear film instability. Increases in TBUT indicate improved tear film stability, while reductions indicate worsening instability. These cutoffs were applied consistently in tables and statistical analyses [25, 26].

#### Quality of life

The Dry Eye-Related Quality-of-Life Score (DEQS) was used to evaluate both symptoms and the impact of DED on participants’ daily functioning over the prior week. The tool consists of two main domains: Ocular Discomfort Symptoms (6 questions) and Limitations in Daily Functioning (9 questions). Each question measured two aspects occurrence and intensity using a 0–4 rating scheme. Occurrence was graded as: 0 = never, 1 = occasionally, 2 = sometimes, 3 = frequently, 4 = constantly. Intensity was graded as: 1 = minimal disturbance, 2 = slight disturbance, 3 = moderate disturbance, 4 = severe disturbance. The overall score was derived using the equation: total score = (sum of all item scores) × 25/(number of items completed), producing a scale from 0 to 100, where greater values indicate more pronounced impairment. Additionally, participants rated overall ocular symptoms and QOL on a 1–6 scale, where 1 represented very good and 6 indicated poor QOL. The DEQS was administered at baseline and again after the 4-week intervention, during the 5th week, in both groups [27].

#### Sample size calculation

Sample size was calculated using MedCalc software (version 12.3.0.0; Ostend, Belgium), based on proportions reported by Branković et al. [16] for severe tear deficiency (Schirmer I ≤ 5 mm): 19.44% in the control group versus 0% in the intervention group. We estimated that 27 participants per group would provide 80% power to determine this difference at α = 0.05 (two-sided). Allowing for approximately 10% attrition, the target enrollment was set at 30 participants per group (total *n* = 60). The MedCalc sample size printout has been included as Supplementary File 1. We acknowledge that using a single prior study as the basis is a limitation; this is noted in the Discussion.

#### Statistical analysis

Data distribution normality and variance homogeneity were assessed utilizing the Shapiro-Wilk and Levene’s tests, respectively, and all assumptions were met. Between-group comparisons of demographic variables were performed using the unpaired t-test. When more than 20% of expected frequencies were < 5, the Chi-square test with Monte Carlo correction was applied. Relationships between qualitative variables were evaluated with the marginal homogeneity test.

The effects of treatment on Schirmer’s test (Schirmer I), tear break-up time (TBUT), and Dry Eye-Related Quality-of-Life Score (DEQS) were analyzed using mixed-model multivariate analysis of variance (MANOVA). MANOVA was selected because these dependent variables are correlated, allowing assessment of the overall effect of polarized light therapy (PLT) while controlling for type I error inflation. When the MANOVA was significant, follow-up univariate ANOVAs were conducted with Bonferroni correction for multiple comparisons. Given the modest sample size, MANOVA was applied cautiously to handle correlated outcomes; results were verified by univariate analyses. Effect sizes were reported as partial eta squared (η²).

All statistical analyses were performed using SPSS software, version 23 (IBM Corp., Armonk, NY, USA), with a significance threshold set at *p* < 0.05. All continuous variables are presented as mean ± standard deviation with one decimal place for consistency; *p*-values are presented to two decimal places (except *p* < 0.001). Analyses were performed on a per-protocol basis, as all participants completed the trial. No dropouts occurred, and therefore intention-to-treat analysis was not required.

## Results

### Comparison between Bioptron and control group according to demographic data

Table (1) displays the demographic characteristics of participants in the Bioptron and control groups. No statistically significant differences were found between the two groups regarding age, BMI, or other general characteristics, with *p*-values ≥ 0.05 as presented in Table (1).

A mixed-design multivariate analysis was performed to evaluate the treatment effects on the measured variables. Statistically significant multivariate effects were observed for the main effect of group, time, and the interaction between group and time. Wilk’s Lambda (Λ) values were 0.35, 0.07, and 0.14; F-values were 34.53, 253.85, and 115.31; p-values were < 0.001 for all; and partial eta squared (ƞ²) values were 0.65, 0.93, and 0.86, respectively.

### Between-groups comparison

At baseline, no statistically significant differences were noted between the Bioptron and control groups in all outcome variables—Schirmer’s test, TBUT, and DEQS—with *p*-values ≥ 0.05, respectively, as shown in Table 2. After one month of intervention, statistically significant differences emerged between the two groups in all measured outcomes, favoring the Bioptron group with *p*-values < 0.05, respectively, as presented in Table (2).

**Table 2:** Within and between group analysis for all outcome variables (*N* = 60) *

Variables	Bioptron Group	Control Group	MD (95% CI)	*p*-value (between groups)	Ƞ^2^
Schirmer’s test (Schirmer I) (mm)					
Pre-treatment	5.3 ± 1.8	5.13 ± 1.6	0.17 (−0.7 to 1.1)	0.71	
Post-treatment	14.87 ± 1.87	6.17 ± 2.2	8.7 (7.64 to 9.76)	0.001^*^	0.8
p-value (within-group)	0.001^*^	0.003^*^			
MD (95% CI)	−9.57 (−10.22 to −8.92)	−1.03 (−1.69 to −0.38)			
TBUT (seconds)					
Pre-treatment	5 ± 1.7	5.2 ± 1.9	−0.2 (−1.2 to 0.7)	0.57	
Post-treatment	10.8 ± 1.62	7.83 ± 2.06	2.97 (1.67 to 4.26)	0.001 ^*^	0.3
p-value (within-group)	0.001^*^	0.001^*^			
MD (95% CI)	−5.83 (−6.52 to −5.15)	−2.6 (−3.29 to −1.91)			
DEQS (score)					
Pre-treatment	69.6 ± 17	69 ± 16.9	0.6(−8.2 to 9.3)	0.9	
Post-treatment	13.7 ± 3.2	40.8 ± 9.9	−24.1(−37 to −17.1)	0.002^*^	0.3
p-value (within-group)	0.001^*^	0.001^*^			
**MD (95% CI)**	55.8(49.6 to 62.1)	28.2(21.9 to 34.5)			

P-value: probability;^*^significance difference; CI confidence interval. MD mean difference, Ƞ^2^ partial eta squared. DEQS Dry Eye-related Quality-of-life Score, mm millimeters, TBUT tear break-up time

### Within-groups comparison

Statistically significant improvements were observed in all outcome measures from baseline to post-intervention in both the Bioptron and control groups (*p* < 0.001), with greater improvements in the Bioptron group (Table 2). MANOVA revealed a significant multivariate effect of PLT compared with control on the combined dependent variables (*p* < 0.001). Subsequent univariate ANOVAs with Bonferroni correction confirmed significant between-group differences in DEQS, Schirmer, and TBUT (all *p* < 0.05).

Quantitative data: According to the data presented in Tables 3 and 4, there were statistically significant improvements in the Bioptron group compared with the control group. These improvements exceeded clinically meaningful thresholds (≥ 10 mm for Schirmer’s test and ≥ 3 s for TBUT). Throughout the intervention period and follow-up, no adverse effects—local or systemic—were observed or reported in either the Bioptron or control groups, confirming the safety and tolerability of photobiomodulation therapy in this population.

**Table 3:** Comparison between Bioptron and control group according to level of schirmer’s test (Schirmer I) and TBUT level

	Bioptron Group (*n* = 30)	Control Group (*n* = 30)	Test value	*p*-value
Level of Schirmer’s test I, *n* (%)				
Before TTT				
Severe tear deficiency	16 (53.3%)	16 (53.3%)	0.0	^MC^1.000
Moderate tear deficiency	14 (46.7%)	14 (46.7%)		
Mild tear deficiency	0 (0.0%)	0 (0.0%)		
Normal value	0 (0.0%)	0 (0.0%)		
**After TTT**				
Severe tear deficiency	0 (0.0%)	10 (33.3%)	53	^MC^ <0.001^*^
Moderate tear deficiency	1 (3.33%)	18 (60.0%)		
Mild tear deficiency	13 (43.33%)	2 (6.7%)		
Normal value	16 (53.3%)	0 (0.0%)		
***p-value#***	^MH^ <0.001**	^MH^ ***0.14***		
**Level of TBUT**,** n (%)**				
**Before TTT**				
Dry eye	16 (53.3%)	18 (60.0%)	0.3	^MC^0.6
Marginal tear film abnormality	14 (46.7%)	12 (40.0%)		
Normal tear film	0 (0.0%)	0 (0.0%)		
**After TTT**				
Dry eye	0 (0.0%)	8 (26.7%)	11.5	^MC^0.003 ^*^
Marginal tear film abnormality	10 (33.3%)	12 (40.0%)		
Normal tear film	20 (66.7%)	10 (33.3%)		
***p-value#***	^MH^<0.001^*^	^MH^0.003^*^		

*P*-value: probability; ^*^significance difference, *DEQS* Dry Eye-related Quality-of-life Score, *mm* millimeters, *TBUT* tear break-up time, *MC* Monte Carlo, *MH* Marginal Homogeneity, TTT treatment

**Table 4:** Comparison between bioptron and control group according to DEQS

QOL, *n* (%)	Bioptron Group (*n* = 30)	Control Group (*n* = 30)	Test value	*p*-value
Before TTT				
Extremely good	0 (0.0%)	0 (0.0%)	0.51	^MC^0.92
Very good	0 (0.0%)	0 (0.0%)		
Good	2 (6.7%)	2 (6.7%)		
Bad	8 (26.7%)	10 (33.3%)		
Very bad	12 (40.0%)	12 (40.0%)		
Extremely bad	8 (26.7%)	6 (20.0%)		
**After TTT**				
Extremely good	10 (33.3%)	0 (0.0%)	30.1	^MC^ <0.001^*^
Very good	16 (53.3%)	6 (20.0%)		
Good	4 (13.3%)	14 (46.7%)		
Bad	0 (0.0%)	10 (33.3%)		
Very bad	0 (0.0%)	0 (0.0%)		
Extremely bad	0 (0.0%)	0 (0.0%)		
***p-value#***	^MH^***<0.001*** ^*^	^MH^***0.01*** ^*^		

*P*-value: probability; ^*^: significance difference; *DEQS* Dry Eye-related Quality-of-life Score; *mm* millimeters, *QOL* quality of life, *MC* Monte Carlo, *MH* Marginal Homogeneity, TTT treatment

## Discussion

The present research investigates the efficacy of BLT in managing DED in postmenopausal women, a condition characterized by disruption of the tear film and inflammatory processes on the eye surface, which significantly impacts QOL [5]. BLT has been shown to increase the expression of anti-inflammatory mediators in tissues and support the restoration of corneal nerve fibers [21]. Patients were recruited and randomly assigned to two groups: the study group received both an education program and BLT, while the control one received the education program only. The aim is to explore innovative treatments for managing DED in this demographic. Both groups had comparable age, weight, height, BMI, years postmenopausal, and eye condition indicating balanced demographic data across the groups.

In our study, the application of BLT for DED demonstrated significant clinical benefits across several measures. The DEQS total scores (symptoms and impact on daily life) decreased significantly in both groups, with a larger reduction in the BLT group compared with control (*p* < 0.001). This finding supports the effectiveness of BLT in alleviating symptoms of dry eye more efficiently than standard care alone. This significant enhancement in symptomatology is echoed in the findings by Arita et al. [28], where IPL-MGX therapy significantly improved lipid layer grade, non-invasive tear breakup time, and reduced symptoms in dry eye patients. Similarly, Vegunta et al. [29] observed that 89% of patients reported improved SPEED2 scores after IPL therapy, underscoring the potential of light-based treatments in managing DED symptoms.

Our study also highlighted a significant increase in tear breakup time (TBUT) post-treatment in the study group, with most participants no longer met TFOS DEWS II diagnostic criteria for DED following treatment, compared to 26.7% in the control group (*p* = 0.003). Before treatment, both groups had similar TBUT levels, with no significant differences. However, the study group showed marked improvement post-treatment, highlighting the efficacy of BLT in enhancing tear film stability. This result aligns with Craig et al. [30], who found that IPL treatment improved lipid layer grade, noninvasive tear breakup time, and visual analog scale symptom scores in a younger patient population, further supporting the benefits of light therapy in improving tear film parameters.

In evaluating tear production through Schirmer’s test, our study found significant improvements in the study group post-treatment, with no participants displaying severe tear deficiency, in contrast to 33.3% in the control group. Furthermore, 53.3% of the study group exhibited normal tear values post-treatment, whereas none in the control group achieved this level of improvement. These results suggest that BLT effectively enhances tear production and mitigates the dry eye symptom severity. Branković et al. [21] similarly reported significant improvement in tear secretion following BLT, particularly in severe and moderate cases. Yan and Wu [31] also found significant improvements in tear secretion in patients treated with IPL, with a gradual decrease in severe tear deficiency rates over time, reinforcing the potential of light-based therapies in enhancing tear production.

Shargorodska et al. [32] conducted an experimental study on rats with anterior surface inflammation and found a significant increase in lacrimation following polarized red-light treatment, indicating a possible anti-inflammatory effect of the therapy. This aligns with our findings, where the study group showed substantial improvements in tear film stability and production.

QOL measures in our study revealed that the study group experienced substantial enhancements in post-treatment, with 33.3% reporting “Extremely good” and 53.3% “Very good” QOL, compared to none in the control group. The control group had a higher percentage of participants reporting “Bad” QOL (33.3%), indicating that the BLT significantly improved the overall QOL for patients with DED. This improvement in QOL is comparable to findings by Yan and Wu [27], who reported higher satisfaction levels in the experimental group treated with IPL, both at 7 days and 30 days post-treatment. Importantly, PLT was well tolerated with no reported adverse events, supporting its preliminary safety profile as a potential therapy for DED. Another limitation of the study is the absence of dry eye subtype classification (ADDE, EDE, or mixed). Future studies should incorporate meibography, tear osmolarity, or additional biomarkers to provide pathophysiologic insight. Also, although the educational program and tear supplementation were standardized across both groups, adherence was not formally monitored. Therefore, compliance differences may have introduced residual confounding. The total satisfaction rates in their study were significantly higher in the experimental group compared to the control group, highlighting the positive impact of light-based therapies on patient satisfaction and QOL. Although our control group received standardized education, the absence of an active comparator (e.g., conventional thermal pulsation or intense pulsed light) limits direct benchmarking of clinical efficacy. Future non-inferiority or head-to-head trials are warranted to define the relative therapeutic role of BLT among established DED interventions.

Several methodological limitations should be acknowledged. First, the relatively small sample size (*n* = 60), based on single prior study, may limit external validity and overestimate effect size. Larger, multicenter trials are needed to confirm and generalize these results. Second, the absence of a sham-light control and the single-blinded design may introduce expectation bias, particularly for subjective measures. Third, adherence was verbally reinforced but not formally monitored, which could further contribute to bias. Fourth, the follow-up period was only four weeks, providing preliminary short-term efficacy and safety data; longer-term studies are needed to evaluate durability of treatment effects. Fifth, objective biomarkers such as tear osmolarity, MMP-9, or meibography were not assessed, and DED subtyping was not performed, limiting detailed mechanistic interpretation. Finally, although both groups received patient education with lifestyle recommendations, their potential confounding effect was minimized by equal application across study arms.

Despite these limitations, our study provides valuable preliminary evidence that BLT is a safe, non-invasive, and well-tolerated adjunctive treatment for DED in postmenopausal women. Future studies with rigorous, double-blinded designs, larger sample sizes, longer follow-up, and incorporation of objective biomarkers are warranted to validate these findings, clarify mechanisms of action, and optimize treatment protocols.

## Conclusion

In this randomized controlled trial, adding Bioptron light therapy to a standardized educational program for 4 weeks led to significant improvements in basal tear secretion (Schirmer I) and tear film stability (TBUT), along with a clinically meaningful reduction in dry-eye-related symptoms, including ocular discomfort, dryness, photophobia, and impact on daily activities as assessed by DEQS. These findings suggest that Bioptron light therapy is a safe, non-pharmacological adjunctive treatment for dry eye in postmenopausal women. Further studies with larger populations, long-term follow-up, and with incorporation of objective biomarkers are essential to validate the sustained safety, effectiveness, and cost-efficiency of this intervention. 

## Data Availability

The corresponding author makes data sets used and/or analyzed in this study available upon reasonable request.

## Declarations

### Clinical Trial Number

NCT05964673.
